# Geographic Disparities in Access to Specialized Dementia Care

**DOI:** 10.1093/geroni/igaa057.2525

**Published:** 2020-12-16

**Authors:** Portia Cornell, Wenhan Zhang, Lindsey Smith, Shekinah Fashaw, Kali Thomas

**Affiliations:** 1 Providence VA Medical Center, Providence, Rhode Island, United States; 2 Brown University, Providence, Rhode Island, United States; 3 PSU School of Public Health, Portland, Oregon, United States

## Abstract

With novel, previously undescribed data on the availability of dementia-specific assisted living communities (ALs), we analyzed variation among counties in the availability of this important service for persons with dementia. In twenty-one states, we identified 6,961 ALs (16%) with a dementia-specific license/certification. Counties with at least one AL providing dementia-specific care had substantially higher college attainment versus counties that had at least one AL, but no dementia-specific beds: 25% versus 18% (p<0.01). Counties with dementia care also had significantly greater median incomes ($54,000 vs. $46,400), and home values ($159,000 vs. $113,000), lower poverty rates (13.7 percent vs. 16.3 percent), and lower proportions of Black residents (7.8 percent vs. 8.7 percent). Our findings are suggestive of a mismatch in need and availability of residential care options for older adults with ADRD that are also low-income or racial/ethnic minorities. Part of a symposium sponsored by Assisted Living Interest Group.

